# Colour Association with Music Is Mediated by Emotion: Evidence from an Experiment Using a *CIE Lab* Interface and Interviews

**DOI:** 10.1371/journal.pone.0144013

**Published:** 2015-12-07

**Authors:** PerMagnus Lindborg, Anders K. Friberg

**Affiliations:** 1 Area of Interactive Media, School of Art, Design, and Media, Nanyang Technological University, Singapore, Singapore; 2 Department of Speech, Music, and Hearing, KTH Royal Institute of Technology, Stockholm, Sweden; Tsinghua University, CHINA

## Abstract

Crossmodal associations may arise at neurological, perceptual, cognitive, or emotional levels of brain processing. Higher-level modal correspondences between musical timbre and visual colour have been previously investigated, though with limited sets of colour. We developed a novel response method that employs a tablet interface to navigate the *CIE Lab* colour space. The method was used in an experiment where 27 film music excerpts were presented to participants (n = 22) who continuously manipulated the colour and size of an on-screen patch to match the music. Analysis of the data replicated and extended earlier research, for example, that happy music was associated with yellow, music expressing anger with large red colour patches, and sad music with smaller patches towards dark blue. Correlation analysis suggested patterns of relationships between audio features and colour patch parameters. Using partial least squares regression, we tested models for predicting colour patch responses from audio features and ratings of perceived emotion in the music. Parsimonious models that included emotion robustly explained between 60% and 75% of the variation in each of the colour patch parameters, as measured by cross-validated *R* ^*2*^. To illuminate the quantitative findings, we performed a content analysis of structured spoken interviews with the participants. This provided further evidence of a significant emotion mediation mechanism, whereby people tended to match colour association with the perceived emotion in the music. The mixed method approach of our study gives strong evidence that emotion can mediate crossmodal association between music and visual colour. The *CIE Lab* interface promises to be a useful tool in perceptual ratings of music and other sounds.

## Background

### Crossmodal association

When associating colour with music, natural soundscapes, or soundscape compositions, do people use different strategies? The question of how associations between visual and auditive modes of perception emerge has been scientifically investigated for more than a hundred years (e.g. [1]). It is generally understood that while some aspects of crossmodal correspondences might have a psychobiological basis [2], other patterns of association might depend on gender [3], be acquired by the individual [4], or defined culturally [5]. The literature identifies at least four mechanisms whereby crossmodal association can emerge, roughly corresponding to levels of neural processing in the brain. An overview is given in Spence’s tutorial [2], which focusses on three classes of crossmodal correspondence: structural, statistical, and semantic. They will be briefly reviewed.

First, at the level of sensory information, crossmodal association can be determined by structural correspondences based on commonalities in the way neurons code sensory stimulation. They occur at early processing stages in the brain. Neuro-biological mechanisms may give rise to crossmodal association, since an increase in stimulus intensity or density generally appears to produce more neural spikes **(**[2], p. 989). The physical structure of the brain might also produce crossmodal capture effects when sensorispecific regions are located in proximity, such as in the deeper layers of the superior colliculus (cf. [6], p. 17).

The second class of crossmodal association is based on the principles of statistical co-occurrence and ecological perception. Stimuli perceived simultaneously through different sensory organs and via parallel neurons might become associated at an intermediate point in the neural processing path if they both increase an organism’s level of alertness or arousal, or if they both happen to have the same effect on emotional state, mood, or affective state ([2], p. 973). In evolutionary terms, such associations may have arisen as part of a strategy for the brain to optimise co-varying sensory input, and then may gradually have become hard-wired ([7], p. 564). The brain’s plasticity is such that these kinds of associations might develop gradually through habits during the life span of a single organism. It is essential for an organism to learn from prior environmental exposure. To determine an optimal action, the information-processing entity must accurately match new sensory input with retained prototypical experiences, as well as, in the case of higher organisms, the appraisal of those encounters through episodic memory ([7], p. 567).

Third, at the cognitive level, crossmodal association is learned consciously. Its effects are available to the individual for inspection, as well as, to some degree, for control and training. Inasmuch as learning can be understood as an individual’s acquisition of methods to decode the communication system of her social environment, this mechanism is based on language. This kind of crossmodal association favours semantically mediated correspondences based on a descriptive terminology that is common between modalities. We believe that this mechanism is deeply connected with listening intentionality, as in Tuuri’s framework [8], though a review goes beyond the scope of the present text.

Beyond structural, statistical, and semantically mediated mechanisms, a fourth way that crossmodal association might arise is via emotion at a pre-cognitive level. The proposition that emotion processing has a role to play in crossmodal correspondences is not new but, as stated in [2], empirical evidence has been lacking. A recent study showed that people’s liking of basic food tastes such as bitter or sweet predicted their crossmodal association of shapes such as angular or rounded with taste ([9]; especially p. 156 suggesting a hedonic mediation effect). In music emotion research, Palmer and collaborators claimed that experimental results provided “clear evidence of cross-modal correspondences based on emotion” ([10], p. 5; see the next section for details). As will be shown, the present results also support an emotion mediation mechanism.

### Previous studies of colour association with music

Research on audiovisual matching has provided evidence that many non-arbitrary correspondences exist between auditory and visual stimulus features. These correspondences have been documented both between simple stimulus dimensions, such as loudness and brightness, and between more complex stimuli, such as visual shapes or pictures, and music (see [2], p. 975). Stimuli features are called ‘modal’ when they identify an aspect that is specific to a single sensory modality, such as the timbre of a sound, or the colour of a light. Features that are not domain-specific are called ‘amodal’. They may reflect more fundamental attributes of stimuli, such as perceived intensity or size. Several studies have lent support to the suggestion that audiovisual correspondences are based on amodal correspondences, for example, the loudness of sound and the ‘lightness’ of light [2].

In the 1930s, Stevens demonstrated that participants paired light grey colour patches with louder sounds and darker grey patches with quieter sounds. More recently, the study by [11] asked participants to match sine waves and colour patches (see also [12] where a similar method was used). The results confirmed the hypothesised correspondence between light intensity and pitch, and between colour hue saturation and loudness. Lipscomb and Kim investigated associations between frequency modulated tones and visual amodal attributes, such as vertical location and size, in a factorial experiment design [13]. They reported strong “pairings” between location and pitch, also known as SMARC effect (Spatial–Musical Association of Response Codes; see [14] and [15]), and between size of visual objects and loudness of tones. They also claimed evidence for higher level and mode-specific correlations: for example, that visual shape was associated with timbre, and that colour matched “equally well with both pitch and loudness” ([13], p. 74), though colours were only reported by word labels.

These studies brought our attention to how people normally speak about aspects of visual colour and how language can function as a mediator of crossmodal association. There has been a long-standing discussion in anthropo-linguistics whether colours are stable, cross-cultural semantic concepts. A meta-study of colour words in 110 languages, including primitive societies, showed that most cultures display concept clusters near ‘red’, ‘green’, ‘yellow’, and ‘blue’ (in addition to ‘white’ and ‘black’) [16]. The authors provided a strong argument that “focal colours” are universal (but for a nuanced perspective by the same researchers, see [17]). Barbière and collaborators [18] investigated the relation between colour and discrete emotions expressed in music, and found that ‘red’, ‘yellow’, ‘green’, ‘blue’ were chosen for ‘happy’ music, and that ‘grey’ (i.e. a de-saturated colour) was chosen for ‘sad’ music. However, the study was limited since it used only four excerpts of classical music and pre-determined word labels for colours (and not actual colour patches). Holm and co-workers [19] investigated the question of colour association with musical genre. They selected 12 colour patches, and music by genre word labels, and found that black was associated with ‘metal rock’, blue with ‘blues’, pink with ‘pop’, and so forth. However, the reason for their selections of audio-visual categories was arbitrary.

In [20], Bresin defined 24 colour patches by sampling parameters in *HSL* (*Hue*, *Saturation*, *Lightness*) at approximately equal distances. This produced a colour palette with arguably more evenly distributed patches, from a perceptual point of view, than in any of the studies mentioned earlier. The participants judged how well the colours matched two different pieces of music that had been performed with twelve different emotional intentions, such as happiness, love or contentment. Bresin analysed the correlations between emotional intentions and ratings of *HSL* colour parameters, and found that colour brightness (i.e. *Lightness*) was associated with positive emotions, and darker colours with negative emotions. While the results are similar to those reported in [18], the stronger methodology lends them greater reliability. A limitation of the study, acknowledged by the author, was that the results appeared to be highly dependent on the different musical instruments used in the stimuli: that is, timbral qualities. Furthermore, since *HSL* is a simple transformation of *RGB* (a device-dependent model where levels of red, green, and blue are added), it gives insufficient information to reconstruct the perceptual appearance of colour patches unequivocally. More important still, the *HSL* dimensions are perceptually confounded, and *HSL* might be described as a pseudo-space. It follows that many statistical operations (e.g. calculating a parametric correlation) are not justifiable on *HSL* values (cf. Method section). The reliance on *RGB* and *HSL* to represent colour is partly the reason why previous research has been limited. Notwithstanding, Bresin’s study is remarkable for having examined patterns of relationship between audio features, emotion, and colour association in a controlled experiment. In the same spirit, Palmer and collaborators [10] investigated colour association with excerpts of music in classicist style. Stimuli were rendered using a MIDI synthesiser, allowing for tempo and tonal mode to be manipulated in a factorial design. They reported that colours of high saturation and *Lightness* (“brighter”), and more towards yellow (“warmth”) were selected for music stimuli in fast tempo, and that conversely, de-saturated (“greyer”), “darker”, and blue colours were selected for music of slow tempo in minor mode. They claimed strong support for emotion as a mediating mechanism for the observed cross-modal associations (see also the previous section). Table 1 sums up the reviewed studies in terms of the methods employed, numbers of stimuli, and specifically, the way in which colours were presented to the participants.

**Table 1:** Overview of methods used in recent studies of colour association with music.

*Authors*	*Year*	*Colour model*	*Number of colours*	*Colour selection*	*Colour presentation*	*Number of sound stimuli*
**Giannakis & Smith**	2001	*HSV*	216	approx. equally spaced	parallel patches	33 (or 72, the description is unclear)
**Datteri & Howard**	2004	*-*	7	arbitrary	parallel patches	8
**Lipscomb & Kim**	2004	*-*	9 (48)	systematic selection	(rating of composites)	48 (tones)
**Bresin**	2005	*HSL*	24	approx. equally spaced	parallel patches	72 (2 pieces x 3 instruments x 12 emotions)
**Barbiere et al.**	2007	*-*	0 (7 words)	arbitrary	parallel word labels	4
**Holm et al.**	2009	*-*	12 (10+b/w)	arbitrary	parallel patches	0 (18 genre labels)
**Palmer et al.**	2013	*HSL*	37	systematic selection	parallel patches	18 (classicist music)
**Lindborg**	2013 (unpublished pilot)	*HSL*	>100,000	quasi-continuous	swatch colour picker	27 (soundscapes)
**Lindborg**	present article	*CIE Lab*	98,553	dimensions perceptually linear, orthogonal & continuous	physical interface (tablet & throttle)	27 (film music)

The studies (where actual colours were used) had three main limitations in regards to colour. Firstly, the number of colour response options was generally small. Some crossmodal effects might not be detected if the response method has low resolution. Secondly, the experimenter’s selection of colour response options was a function of the representation scheme rather than a perceptual model. Analytical results might not be ecologically valid if the colour model does not match human perception. Moreover, it is difficult to replicate experiments where colours have not been specified unambiguously. Thirdly, the experiments presented patches in parallel or in Mondrian-style patterns, that is, there were multiple response options side by side, and the respondent might indicate one by clicking on it. This approach introduces two problems. One is that low-high and left-right screen placement might cause spatial bias (SMARC effect; [14]). The other is that simultaneous presentation is known to cause contrast effects [21]. Since the eye detects colour not only at the focal point but also in a large peripheral region [22], the appearance of a colour patch depends on adjacent patches. As we will show, these sources of potential bias can be avoided by designing the response interface accordingly.

## Aims

We are interested in identifying which aspects of music might cause certain colours to be selected, what perceptual or cognitive mechanisms might be involved, and whether emotion plays a significant part in crossmodal correspondences. First, we formulated three testable questions: *Do people associate different colours with music expressive of discrete emotions*? *Do colour associations align with perceived dimensional emotions in music*? *Do men and women differ in colour patch association with music*? Then, we explored the extent to which colour association could be explained by computationally extracted audio features and emotion ratings of the music: specifically, whether emotion would contribute to predicting colour over and above audio features. Finally, we aimed to probe the perceptual and cognitive mechanisms involved in crossmodal correspondence, from music via emotion to colour, through a qualitative analysis of focus interviews.

## Methods

### Response interface and *CIE Lab* colour space

As seen in the Introduction, previous studies might have been biased due to colour representation schemes having low resolution and uncertain congruity with human perception, and due to the simultaneous presentation contrast effect. To avoid these problems, we designed a novel response interface from scratch.

To be able to detect effects of crossmodal correspondences between timbral features of music and visual colour, which might be small, the response interface needed to present a large range of colours with high resolution. To assure ecological validity of the captured data, we needed a colour representation model that closely matches human perception. CIE L*a*b* (henceforth *Lab*) meets these requests (see [23] and [24] for details). This colour model has three orthogonal dimensions, developed to match human perception, that are labelled *Lightness* (also referred to as L*; dark-to-bright), a* (green-to-red), and b* (blue-to-yellow). Note that because colour perception is determined by contextual factors, a minimal requirement for a *Lab* specification to be meaningful is that the reference illuminant or ‘white spot’ is indicated. The dissimilarity between colours can be calculated as Euclidian distance in *Lab* space. By extension, arithmetic operations can be performed on *Lab* representations, such as calculating the mean colour of a set of colour patches.

Various possible hardware interfaces were considered to navigate *Lab* space. We wanted to prioritise giving the user access to as large a range of colour as possible. It was also of interest to include the size of the colour patch as another response channel, since previous research [13] had reported a strong pairing between visual size and the loudness of tones.

**Fig 1:**
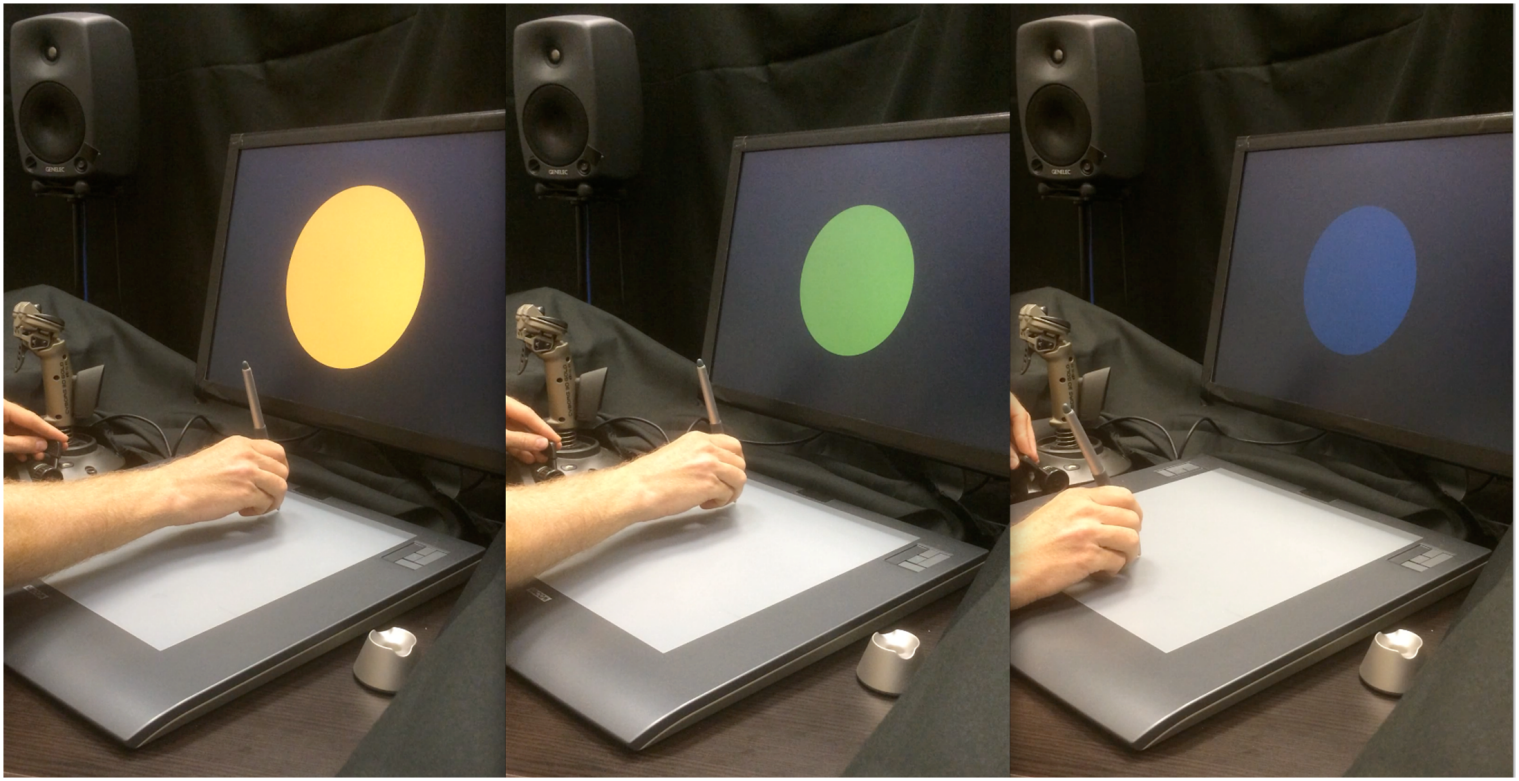
Three photos of the response interface being manipulated.

To solve these design requirements, we opted to present the user with a single colour patch whose appearance could be continuously manipulated. It is defined by four parameters—size (*Size*) and colour (*L*, a*, b*)–that are mapped from two standard input devices, a Wacom tablet and a USB joystick throttle. The user holds the Wacom pen in the dominant hand and manipulates the throttle with the other. The (x, y) contact point of the pen on the tablet is mapped to a point (a*, b*) in Lab space, and pen pressure is mapped to the radius of the colour patch. By pressing the pen down the patch increases in size, and when the pen is lifted it disappears from the screen. The joystick throttle is mapped to *Lightness*. The software was written in Max [25]. See Fig 1 for photos of the interface setup. The response channels (pen pressure, throttle, tablet x, tablet y) have high physical and temporal resolution. Parameter mappings are linear, continuous, and separate, i.e. the four physical response dimensions are not confounded in relation to the four perceptual dimensions, which allows the user to quickly and intuitively learn how move in the space and select a colour patch response.

A design challenge arises with *Lab* in this context since the physical response input space is conceptually a box in four dimensions (*Size*, *Lightness*, a*, b*) but the effective output space consists of cells that may have either a visible or an invisible colour. The latter are colours produced by tristimulus combinations that are not perceptible by the human visual system. To cope with this situation, we created a hybrid space where cells with invisible colour are given the colour of the closest cell with visible colour. As a result, navigating the whole input space is meaningful, so that, for example, the user can move the pen across the entire square-shaped tablet. For the software used in the present experiment, the hybrid space was pre-calculated with a resolution of two *Lab* units, yielding 98,553 different colours. (In a newer version, pre-calculation has been replaced by direct calculation of colour space transformations using formulæ from [23], yielding fully continuous mapping between physical interface and *Lab* space.) The hybrid space had 51*101*101 = 520,251 cells, but since most (~81%) did not correspond to a visible colour, they 'borrowed' the nearest true colour from the true *CIE Lab* space.

To our knowledge, the integration of *CIE Lab* with a physical response interface has not previously been described in published perception research. We believe that its large gamut of colour, available for real-time manipulation, represents a novel approach to research in crossmodal association between colour and music. The interface was employed in an investigation of colour association with a selection of music stimuli for which the affect had been validated in a previous study. Computational musical and psychoacoustic features were extracted with commonly available tools.

### Selection of music stimuli and extraction of audio features

In [26], Eerola and Vuoskoski derived a set of 110 music excerpts from a large collection of typical film music that was largely unfamiliar and ecologically valid from a non-expert listening point of view. The set was rated on scales of discrete emotion (which they labeled as *Anger*, *Fear*, *Happy*, *Sad*, *Tender*) and dimensional emotion (*Valence*, *Energy*, *Tension*), as well as two additional scales (*Beauty*, *Liking)* intended to make it possible to control for effects of individual preference towards music of varying complexity (cf. [9] about the effect of liking in a different context). It was used in [27] in an experiment aimed at validating perceptual features in music information retrieval. For the purpose of the present work, we created an abbreviated version that would span the range of both dimensional and discrete emotions, by selecting excerpts representative of the extremes on the rating scales. For each of the discrete emotions, two files were chosen that had been rated as very high on that scale and at the same time low on the other four discrete emotion scales. For each of the dimensional scales, two files were picked at the very high end and two at the very low end of that scale, which at the same time had been rated at the opposite end on the other dimensional scales. For example, to pick files for the discrete emotion *Anger*, we calculated:

max (*Anger*—max (*Fear*, *Happy*, *Sad*, *Tender*));

and for the dimensional emotion *Valence* at the high extreme, we calculated:

max (*Valence*—max (*Energy*, *Tension*));

and for *Valence* at the low end of the scale:

min (*Valence*—min (*Energy*, *Tension*)).

Similarly, files for *Beauty* and *Liking* scales were selected, to yield a collection of 27 stimuli. The scores for *Beauty* and *Liking* were strongly correlated (*r* = 0.94), and it therefore made sense to simplify by merging them in a variable called *Preference*. Using this variable, two files representing high (i.e. very beautiful, very liked) and two files representing low *Preference* were picked. The median scores of the selected excerpts by extreme group are indicated in Table 2. During the trials, the experimenter found that the same music appeared in two original excerpts (indices 49 and 101). This appears to have been missed by the researchers who previously worked with the full set, because the two had been rated separately in both [26] and [27]. Since separate colour associations were obtained in our present experiment, both excerpts were retained in the analysis.

**Table 2:** Colour response parameters to music excerpts expressive of five discrete emotions.

	*Anger*	*Fear*	*Happy*	*Sad*	*Tender*
***Emotion rating***	6.39	7.105	7.775	6.78	6.615
***Size***	0.609	0.556	0.577	0.521	0.507
***Lightness***	43.2	37.2	58.1	40.8	50.0
a*	27.5	15.1	14.4	9.1	5.6
b*	22.5	2.2	31.4	-6.7	-11.2

*Emotion rating* = relative strength of emotion in music stimuli in range [1…9]. *Size* = relative size of colour patch in range [0…1]. *Lightness* = *CIE Lab Lightness* (dark-to-bright) in range {0…100}. a* = CIE Lab a* (green-to-red) and b* = CIE Lab b* (bleu-to-yellow), both in range [-100…100]. *Lab* values calculated with D65 as illuminant. See Methods section for details.

The stimuli were normed for loudness with a method that is common in broadcast media (ITU-R BS.1770-1) using a Matlab script [28]. Computational low- and mid-level musical features (see [27] and [29] for a discussion of the hierarchy of such features) of the excerpts were extracted using the *MIR Toolbox* [30], and psychoacoustic descriptors were produced with *Psysound3* ([31], [32]). The musical features were grouped in three categories: Rhythmic & Dynamic *(Tempo*, *Attack time*, *Lowenergy);* Timbral *(Spectral centroid*, *Brightness*, *Spectral spread*, *Rolloff [85%]*, *Spectentropy*, *Spectral flatness*, *Irregularity*, *Zerocross rate*, *Spectralflux);* and Tonal *(Chromagram peak position*, *Keyclarity*, *Mode)*. For each feature, the mean across successive time frames yielded a single representative value. The psychoacoustic descriptors were: *Loudness* (N), *Sharpness* (S), *Roughness* (R) and *Fluctuation strength* (F). For each descriptor, the median of values in successive time frames yielded a single representative value. See [33] and [31] for details on the psychoacoustic models, and [34] for a work where we have employed these descriptors. In the present work, *Fluctuation strength* was estimated using a unitless function in the *MIR Toolbox*. Because musical features and psychoacoustic descriptor stem from different research traditions, the terminologies and methods are slightly different. For convenience, we refer to both as ‘audio features’ in the present work. Detailed interpretations are provided in the Results section, as and when they emerged in significant correlations with colour patch parameters.

### Analysis methods

The analysis was carried out in three parts. Relevant methods will be briefly discussed here, before moving on to presenting the procedure and results. Statistical analyses were carried out in *R* [35].

In the first part of the analysis, descriptive statistics and a priori tests were performed on the parameters of colour patch associations to find differences in responses between discrete emotions, and between high and low dimensional emotions. Only the stimuli that had been previously selected as representatives of the extremes of the emotional scales were included here. This analysis was made using two-sample non-parametric tests. In the second part of the analysis, we included all the available data. We first explored patterns of relationships between the parameters of colour patch associations and the audio features (cf. [36]). We then investigated the role of emotion as a mediating variable between audio features and colour patch parameters by comparing different models using partial least squares regression (*PLS*; [37], as implemented in [38]). *PLS* is a method for making inferences about the relationship between causally related blocks of variables in terms of unobserved latent factors (in our case, the emotion ratings). In the third part of the analysis, spoken responses from the focus interviews were subjected to classical content analysis (for reviews, see [39] and [40]), following [41].

### Ethics statement

The research project, including behavioural data collection and participant interviews, received approval by the Institutional Review Board of Nanyang Technological University, Singapore, reference: IRB-2014-08-010. An open call for participation was broadcast to a large number of people via email and social media. Those who expressed an interest were invited to an individually scheduled meeting. A written document detailing the research project and the experimental procedure was provided. Before starting, the text was discussed and clarifications made when needed. It was highlighted that the experiment would carry minimal risk, and that the participant was free to discontinue at any point. In each case, she/he agreed to pursue with the experiment. Her/his name and contact address were recorded and later transferred to a separate file. Due to an oversight, the participant was not at this point asked to sign the Consent Form. This transpired only after the end of the experiment sessions. The participants were then contacted individually, whereupon each one gave their written consent, retrospectively.

## Perceptual Experiment

### Apparatus, participants, and procedure

An experimental setup was created in an office space with carpeted floor, bookshelves along two walls, and a 4 m^2^ rug on one wall. The ambient noise level was 33 dBA (50 dBC) as measured with a calibrated SPL meter (Extech 407090). The reverberation time was comfortably short, RT60 ≈ 0.8 s as measured by a simple tool (AudioTools on an iPhone). The colour reproduction of the screen (Apple 22” LCD) was calibrated using a Spyder4 system. Window daylight was blacked out with thick curtains, and fluorescent tubes created a uniform ambient light. The participant was comfortably seated with the head 70 cm from the screen, whence the colour patch had a maximal apparent diameter of 18°. The peripheral colour of the screen was black. To minimise visual distraction, black cloth was arranged in an area around (±75°) and above (±45°) the screen. This created a dark background with no cluttering objects within the field of vision (see Fig 1). For sound playback, a 2.1 system was used, consisting of two Genelec 8030 full-range near-field monitors and a 7050a subwoofer. The subwoofer crossover frequency and level relative to the monitors were adjusted so that playing white noise through the system produced identical readings on dBA and dBC scales (± 1 dB). The music excerpts (normed for loudness as described earlier) were played back at a uniform amplification level throughout the experiment, producing a sound pressure level between 71 and 79 dBA at the listening position.

The participants (n = 22, nine females) were recruited from a school of art, design, and media. They were university students (8), young professionals (2), academic faculty (7), and other staff (5). The sample might be biased in that these participants were likely to be more attentive to visual design than the larger population. Median age was 30 years old, in a range between 22 and 55. Each participant was offered 10 SGD as a token of appreciation.

After the purpose, duration, and procedure had been explained to the participant, she was given some time to familiarise herself with the interface, and to do a trial run with music to get used to the sound level. The stimuli were played in randomised order, with a short silence between each. The participant continuously manipulated the colour and size of the patch on the screen using the physical interface, as described in the Method section. After completing all stimuli, the experimenter conducted a short interview organised around four previously set questions.

### Data preparation

**Fig 2:**
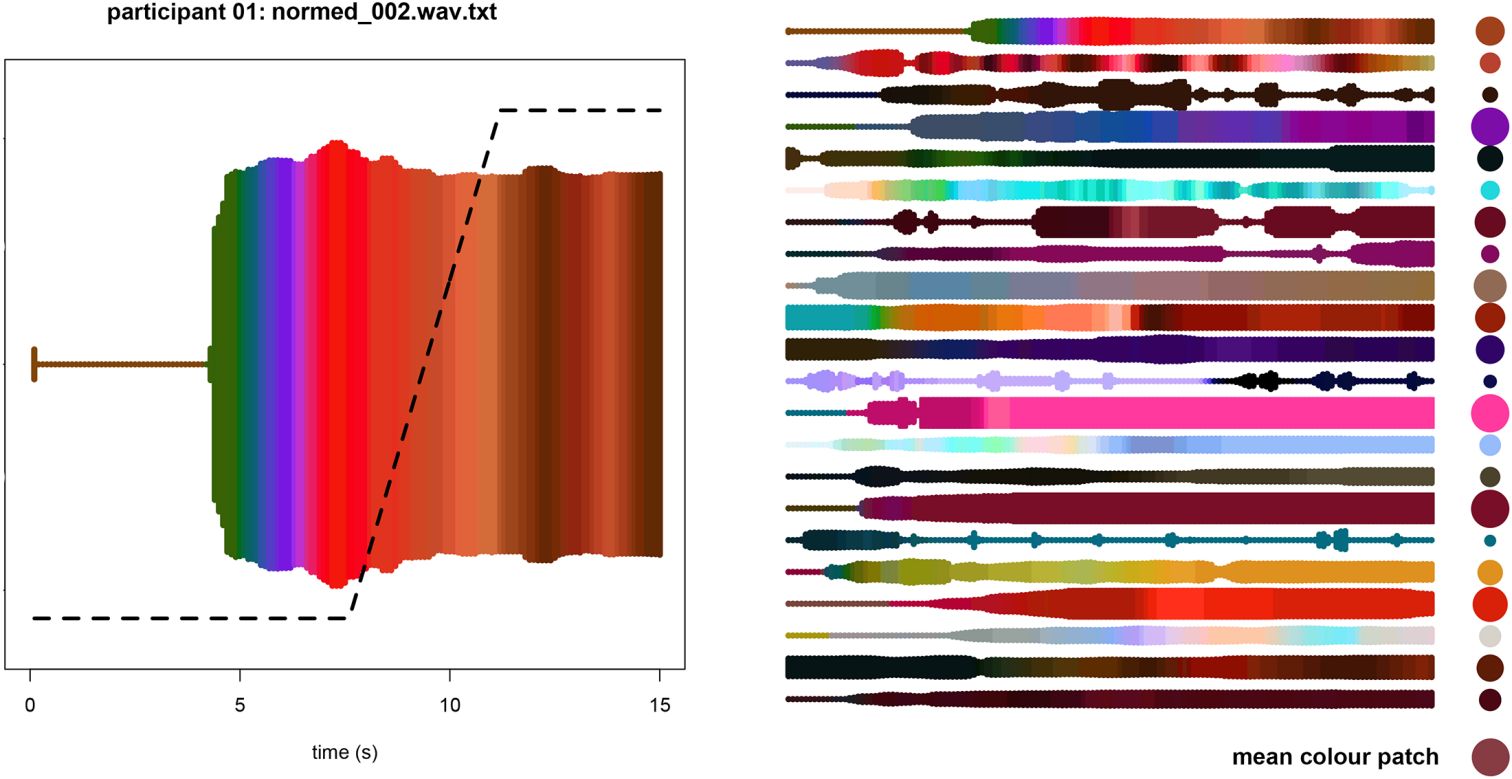
Illustration of how colour response data were weighted and averaged. The diagram to the left shows the envelope of the colour patch association over the course of one stimulus by one participant, overlaid with the weighting curve. To the right is the stack of ‘colour trails’ for the same stimulus by all the participants. To the very right is a column of filled circles representing the weighted average colour patch for each participant, and below it is the overall, mean colour patch association for the stimulus.

The participants had been instructed to select a single colour and size to match each music stimulus. When a new excerpt started playing, the experimenter observed that the participant typically waited a couple of seconds, sometimes ‘searching around’ in the colour space, presumably until the character of the music had stabilised in their mind and a meaningful colour association had become possible for them to express. This was clear from the focus interviews. One participant explained: “once the music starts playing, I spend a second or so to find a starting colour”. Another described the process as “I listened to the music and then first thing is: I like or not? …[I] translate in my mind: what is this music? Then I choose the colour”. A third person said he would “hear the music, then choose the colour that corresponds to the feeling that [I] feel from the music. For some music immediately, for some [it] is more complex” (Participants 19, 17, and 11, respectively; see below for further results from the qualitative analysis of interviews). After deciding on a colour response, they would typically hold on to it more or less steadily (as instructed) until the music excerpt ended. Fig 2, left diagram, illustrates a typical development of colour association over the course of a 15-second music excerpt. There is little previous research probing the perceptual mechanisms involved in this kind of colour response. One study [42] reported that listeners gain knowledge about various aspects of music, including mood, from excerpts that are 3 seconds or shorter. However, their experimental procedure was based on retrospection and free-form response without time constraints, which is different from the present study, where participants responded simultaneously with the stimulus. By looking at the *Size* response envelopes in our present data, we observed that participants sometimes waited several seconds before even putting the pen to the tablet, and typically took 5 seconds or more before settling on a colour. It can be assumed that some of the waiting and searching was conditioned on knowing that each stimulus lasted around 15 seconds. From inspecting the data, it was clear that the final part of the colour response envelope was the most reliable representation of the association that the participant had made to the music excerpt as a whole. With this in mind, we constructed an algorithm to derive a single point (*Size*, *Lightness*, a*, and b*) from the response envelopes, as follows. The response channels were sampled at 10 Hz, producing around 600 values for each stimulus. The first half was discarded (i.e. values were given zero weight). From the middle to the three-quarter point, weighting values were incremented linearly, until reaching a maximum that was held constant until the end. See Fig 2 (left diagram) for an illustration of the weighting curve. The weighted *Size* and *Lab* averages for each stimulus and participant represented the colour patch association. As illustrated in Fig 2 (right diagram), these could then be averaged across participants to yield a mean colour patch for the stimulus.

### Agreement

The rating agreement among the participants was measured by Cronbach’s α. Agreement was good for *Lightness* (α = 0.74) and acceptable for *Size* (α = 0.58) and b* (α = 0.67), but poor for a* (α = 0.42). It was found that responses on the a* parameter (green-to-red) by three participants were negatively correlated with the mean of the others, and we therefore excluded them. After this, the agreement among the remaining 19 participants was good for *Lightness* (α = 0.70) and acceptable for *Size* (α = 0.59), a* (α = 0.60), and b* (α = 0.65).

## Results with Discussions

### Part one: Planned tests

#### Discrete emotions

*Do people associate different colours with music expressive of different discrete emotions*? Two excerpts had been selected to represent each of the five discrete emotions, each with a score (as reported in [26]) near the maximum on one specific discrete emotion scale and relatively low scores on others. Each extreme was represented by 2 music excerpts rated by 19 participants; there were thus 38 values for each variable (*Size*, *Lightness*, a*, and b*). The results are given in Table 2.

**Fig 3:**
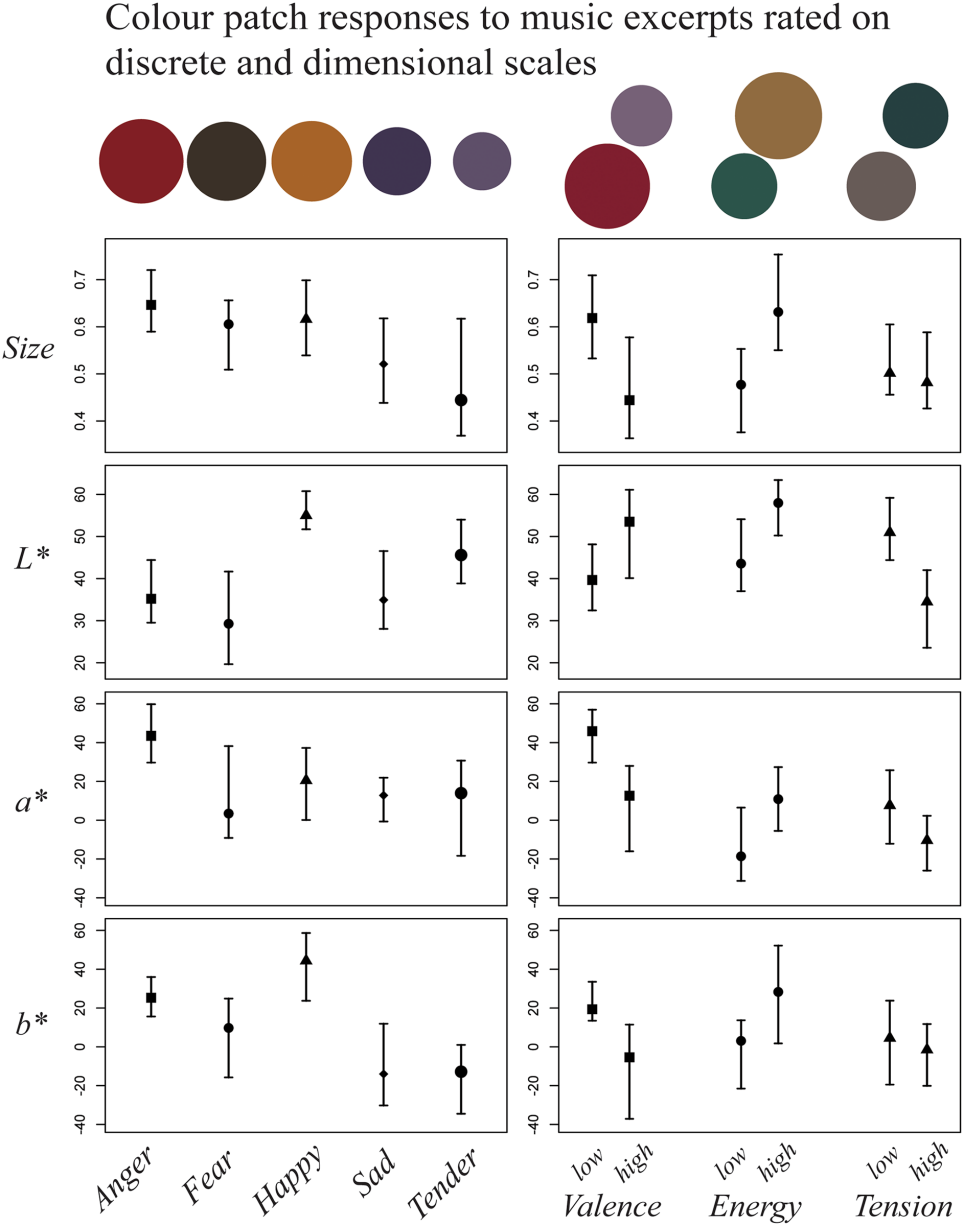
Colour patches and error bars for *Size*, *Lightness*, a* (green-to-red), and b* (blue-to-yellow) for film music excerpts expressing five discrete emotions and low or high dimensional emotion. Colour patch ranges as in Table 2. Median values with bootstrapped 95% confidence intervals around the median (5000 simulations each). Note that the colour plots are for illustration only. Their appearance might be approximate, as colour reproduction depends on encoding, screen setting, and so forth. For patch size, see the Procedure section.

All distributions were assessed with Shapiro-Wilks test. Since 5 (out of 20) were non-normal (p < 0.05), the non-parametric Kruskal-Wallis rank sum test was chosen to test for differences in colour parameters between emotions. *Size*, *Lightness*, a*, and b* were in turn taken as the dependent variable. In each test, the null hypothesis was that all medians of responses by group were equal; the alternative was that at least one group’s median differed from the rest. As can be seen in Table 3, the tests revealed significant differences on each colour patch parameter. Therefore, the differences between each of the five discrete emotions were tested using Tukey’s Honest Significance method, with familywise error rate at α = 0.05. See also the plot of error bars in Fig 3.

**Table 3:** Results from tests on differences in colour association parameters between discrete emotions.

	*χ* ^*2*^	*p*	*HSD*
***Size***	9.8	0.044*	–
***Lightness***	24.6	0.0006***	Happy > Anger **, Happy > Fear ***, Happy > Sad **
a*	12.5	0.014*	Anger > Tender **
b*	33.2	1.1e-06***	Anger > Sad **, Anger > Tender ***, Happy > Fear *, Happy > Sad ***, Happy > Tender ***

χ^*2*^ = Kruskal-Wallis statistic. HSD = Tukey’s Honest Significant Difference, controlling for familywise error rate. *p* = probability of obtaining a test statistic result at least as extreme as the one that was actually observed, under the assumption that the null hypothesis (no effect) is true. Asterisk codes for degree of significance: *** p<0.001; ** p<0.01; * p<0.05. For explanation of variable names, see Table 2.

The results showed that that in the present experiment, *Happy* music was associated with significantly lighter colours than *Anger*, *Fear*, and *Sad* music, and at the same time, with more yellow (rather than blue) colours than *Tender*, *Fear*, and *Sad* music. Similarly, *Anger* music was associated with more yellow colours than *Tender* and *Sad* music, and with more red (rather than green) music than *Tender*. No other discrete emotion difference was significant under this test. Some effect sizes (Cohen’s *d*) were fairly large, such as the difference between *Happy* and *Sad* music, 0.74 SD for *Lightness*, and 0.90 SD for b* (‘yellowness’).

#### Dimensional emotions

*Do colour associations align with perceived dimensional emotions in music*? Two excerpts had been selected to represent low and high *Valence*, *Energy*, and *Tension* (with ratings from [26]). As for the discrete emotions, the extremes were represented by 38 values for each variable (*Size*, *Lightness*, a*, and b*). The results are given in Table 4.

**Table 4:** Colour response parameters to music excerpts expressive of high and low dimensional emotions.

	*Valence*		*Energy*		*Tension*	
	*low*	*high*	*low*	*high*	*low*	*high*
***emotion rating***	2.7	7.4	2.9	7.8	2.6	7.8
***Size***	0.590	0.401	0.458	0.589	0.495	0.475
***Lightness***	41.1	53.5	46.5	60.5	54.9	34.5
a*	39.5	10.7	-9.6	8.7	7.7	-9.2
b*	18.8	-5.4	-2.3	17.9	7.6	-1.4

Emotion and colour patch ranges as in Table 2. For explanation of variable names, see Table 2.

The distributions were assessed as before. Since 2 (out of 24) were non-normal, the non-parametric Wilcoxon rank test with continuity correction (a.k.a. Mann-Whitney U test) was employed to compare colour patch associations between *high* and *low* stimuli. The null hypothesis of this test is that two vectors do not differ by more than an arbitrarily small location shift. In other words, the test was whether music excerpts with high and low dimensional emotion, respectively, had been associated with patches of the same size and colour, or different. The results are given in Table 5. See also the plot of error bars in Fig 3.

**Table 5:** Results from tests on differences in colour association parameters between high and low dimensional emotions.

	*Valence*			*Energy*			*Tension*		
	*W*	*p*	*d*	*W*	*p*	*d*	*W*	*p*	*d*
***Size***	263	0.0061**	-0.39	764	0.0013**	0.47	493	0.99	-0.05
***Lightness***	609	0.19	0.27	811	0.0001***	0.52	175	0.00009***	-0.78
a*	263	0.0024**	-0.59	658	0.057	0.42	363	0.13	-0.36
b*	252	0.0039**	-0.65	716	0.0091**	0.48	414	0.35	-0.24

***W*** = Wilcoxon test statistic. Probability codes as in Table 2. ***d*** = effect size (Cohen) in standard deviations. For explanation of variable names, see Table 2, and for asterisk codes of p-values, see Table 3.

The difference between colour associations with stimuli of low and high *Valence* was expressed in *Size*, a*, and b*; effect sizes were medium (Cohen’s d = 0.39, 0.59, and 0.65 SD, respectively). Low-valence music (e.g. perceived as unpleasant) was associated with larger colour patches towards red and yellow. The difference between low and high *Energy* stimuli was expressed in *Size*, *Lightness*, and b*, with medium effect sizes (0.47, 0.52, and 0.48 SD, respectively). Low-energy music (e.g. perceived as calm or boring) was associated with smaller, darker colour patches towards blue. The difference between low and high *Tension* was expressed in *Lightness* with a fairly large effect size (0.78 SD). Low-tension music (e.g. perceived as easy-going) was associated with lighter colours.

#### Preference and gender

Colour association in relation to *Preference* (i.e. the average of *Beauty* and *Liking* scores from [26]) were investigated in the same way as for dimensional emotions. There were no statistically significant differences in colour patch associations between music of low and high *Preference*.

Finally, we investigated colour association in relation to gender. There were 11 males and 8 females, and the group size difference was non-significant (Pearson’s χ2 = 2.9, p = 1 n.s.). The distributions of colour response parameters were tested as before and several were found to be non-normal. Therefore, the two-sample Wilcoxon test with continuity correction was chosen to test colour patch differences between male and female participants. Note that this test allows the two compared vectors to be of different lengths. Analysis revealed that the female participants generally made colour patches with smaller *Size* (W = 19938, p = 2e-13***). The effect was of medium size (d = 0.69 SD). If true, this might indicate a gender difference of relevance to understanding how the ‘sound to physical size’ association works, generally believed to be a deep-seated crossmodal correspondence based on the ecological principle.

### Part two: Predicting colour from audio features and emotion

We proceeded with correlation and regression analyses including all the available data. In a first step, we correlated the colour patch parameters averaged across participants against the previously extracted audio features of the music stimuli and colour patch parameters. All variables were power transformed using functions in [36], resulting in normal distributions (p < 0.1). Thus a parametric correlation method could be applied, and results are shown in Table 6.

**Table 6:** Correlations between colour patch parameters and audio features in 27 film music stimuli.

		*Colour Patch Parameters*			
*Type*	*Variable*	*Size*	*Lightness*	a*	b*
*Psychoacoustic*	***Loudness***				
*Psychoacoustic*	***Sharpness***	0.63***		(0.48*)	(0.56**)
*Psychoacoustic*	***Roughness***				
*Psychoacoustic*	***Fluctuation strength***	(0.44*)			
*Rhythmic & Dynamic*	***Tempo***				
*Rhythmic & Dynamic*	***Attack_time***	(0.5**)	(0.44*)		(0.4*)
*Rhythmic & Dynamic*	***Lowenergy***		(0.39*)		
*Timbral*	***Spectral centroid***	(0.53**)		(0.44*)	(0.51**)
*Timbral*	***Brightness***	0.63***		(0.44*)	(0.59**)
*Timbral*	***Spectral spread***			(-0.42*)	
*Timbral*	***Rolloff (85%)***	(0.6**)		(0.51**)	(0.51**)
*Timbral*	***Spectentropy***	0.69***		(0.61***)	(0.57**)
*Timbral*	***Spectral flatness***	(-0.45*)		(-0.52**)	
*Timbral*	***Irregularity***		(0.41*)	(-0.43*)	
*Timbral*	***Zerocross rate***	(0.53**)			(0.57**)
*Timbral*	***Spectralflux***	0.73***		(0.42*)	(0.61***)
*Tonal*	***Chromagram (PeakPos)***			(0.43*)	
*Tonal*	***Chromagram (Centroid)***				
*Tonal*	***Keyclarity***		(0.56**)		
*Tonal*	***Mode***		(0.41*)		

Pearson’s *r* on Box-Cox transformed variables. Comparisons significant at the familywise error rate corrected level (*α*_c_ = 0.00064) are given without parentheses. Comparisons where *α*_c_ < p ≤ 0.05 are included in parentheses. Comparisons with p > 0.05 are omitted for clarity. For explanation of variable names, see Table 2, and for asterisk codes of p-values, see Table 3.

#### Correlations

With familywise error rate set at *α* = 0.05, the significance of each comparison was evaluated at the *α*_c_ = 0.00064 level (Dunn-Sidak’s correction for 80 comparisons). *Size* correlated significantly with *Sharpness*, *Brightness*, *Spectentropy*, and *Spectralflux*. Both *Sharpness* and *Brightness* are related to spectral shape, specifically, the amount of high-frequency energy. A high *Spectentropy* indicates that energy is randomly distributed (i.e. with high uncertainty), such as in a sound with a wide spectrum and few peaks ([30], p. 157). A high *Spectralflux* results from audio with a high degree of spectral change between consecutive analysis windows, i.e. timbral variability over time ([30], p. 58). The relationship between colour patch *Size* and these features, if true, might be due to a crossmodal effect at the structural level. Consider a bright-sounding music with a high degree of random variations (perhaps from shrill, fluttering high-range instruments). Such a sound would attract the listener’s attention and be perceived as having a ‘larger spectrum’. This property might be transferred to an increased physical size of a visual object via the ecological correspondence mechanism.

The *CIE Lab* parameters did not correlate significantly with audio features at the strict level set by the familywise error rate. We will nevertheless discuss a few cases where colour might correlate more weakly with audio features, at an uncertain level of significance. Thus, *Lightness* might be associated with features such as timbral *Irregularity* and the tonal features *KeyClarity* and *Mode*. The first represents the degree of variation of successive harmonic peaks in the spectrum (this would indicate a timbrally rich music); the second, the relative strength of a tonal centre (e.g. a stable, predictable music); and the third, the degree of minor-to-major tonality, so that a higher value is ‘more major’ (see [30], p. 112, 127, and 129). All three can be understood as descriptors of how clearly defined the music is in terms of timbre and tonality. *Lightness* might also correlate weakly with *Attack time*, which is a descriptor of how sharp the dynamic variations are (i.e. note onset; see [30], p. 99). The influence of these features on *Lightness*, if true, might originate in a crossmodal (amodal) association effect linked to the semantic concept of ‘clarity’, so that ‘unclear’ music is associated with darker and more shadowy colours. The results involving *Lightness*, though not statistically significant at the *α*_c_ level in the present data, replicate reported findings in [10] and [20].

CIE a* (green-to-red), similarly to *Size*, was correlated with *Spectentropy* and several other timbral parameters, but might differ by being in addition weakly and negatively correlated with *Irregularity* and *Spectral spread*, as well as with *Chromagram peak position*. The latter is the unwrapped chroma, and thus depends not only on pitch class (as is the case with *Chromagram centroid)* but also on tonal register ([30], p. 118). Therefore, its correlation with a*, if true, might depend on spectral shape in ways similar to *Brightness* and other timbral features, rather than absolute key.

Finally, CIE b* (blue-to-yellow) might differ from *Lightness* in terms of how it is influenced by *KeyClarity* and *Mode*, and from a* in that it did not appear to be influenced by certain audio features, such as *Irregularity*, *Spectral flatness*, and *Spectral spread*.

#### Regressions

In a second step, we analysed the prediction of colour patch association variables from independent and potentially mediating variables using multivariate partial least squares regression (*PLS*; following examples in [38]). The *PLS* algorithm finds the optimal linear combination of all potential predictors and projects this combination onto a space of lower dimensionality (in our present case, two components) that explains a maximum of the variance in the matrix of dependent variables (in our case, *Size*, *Lightness*, a*, and b*). Three prediction models were defined in order to evaluate the mediating influence of emotion on colour association with music. In the first, only audio features were included in the pool of potential predictors. In the second and third models, ratings on dimensional and discrete emotion scales, respectively, were added to the pool. In each model, we limited the number of components that the PLS regression could consider to two. This was a reasonable size since the number of cases was 27 in the present data. Increasing to three components might be justifiable, but when testing it, it did not produce significantly better results, and therefore the smaller model was preferred. Note that models two and three differed from the first and more basic model only in having access to more information initially, i.e. the emotion ratings. However, the complexity of all three models was the same, as each would yield two components employed to simultaneously predict the four dependent variables. If the emotion ratings did not contain information capable of providing additional explanatory strength, then there would be no difference when going from the basic first model, with audio features only, to the extended second and third models.

**Fig 4:**
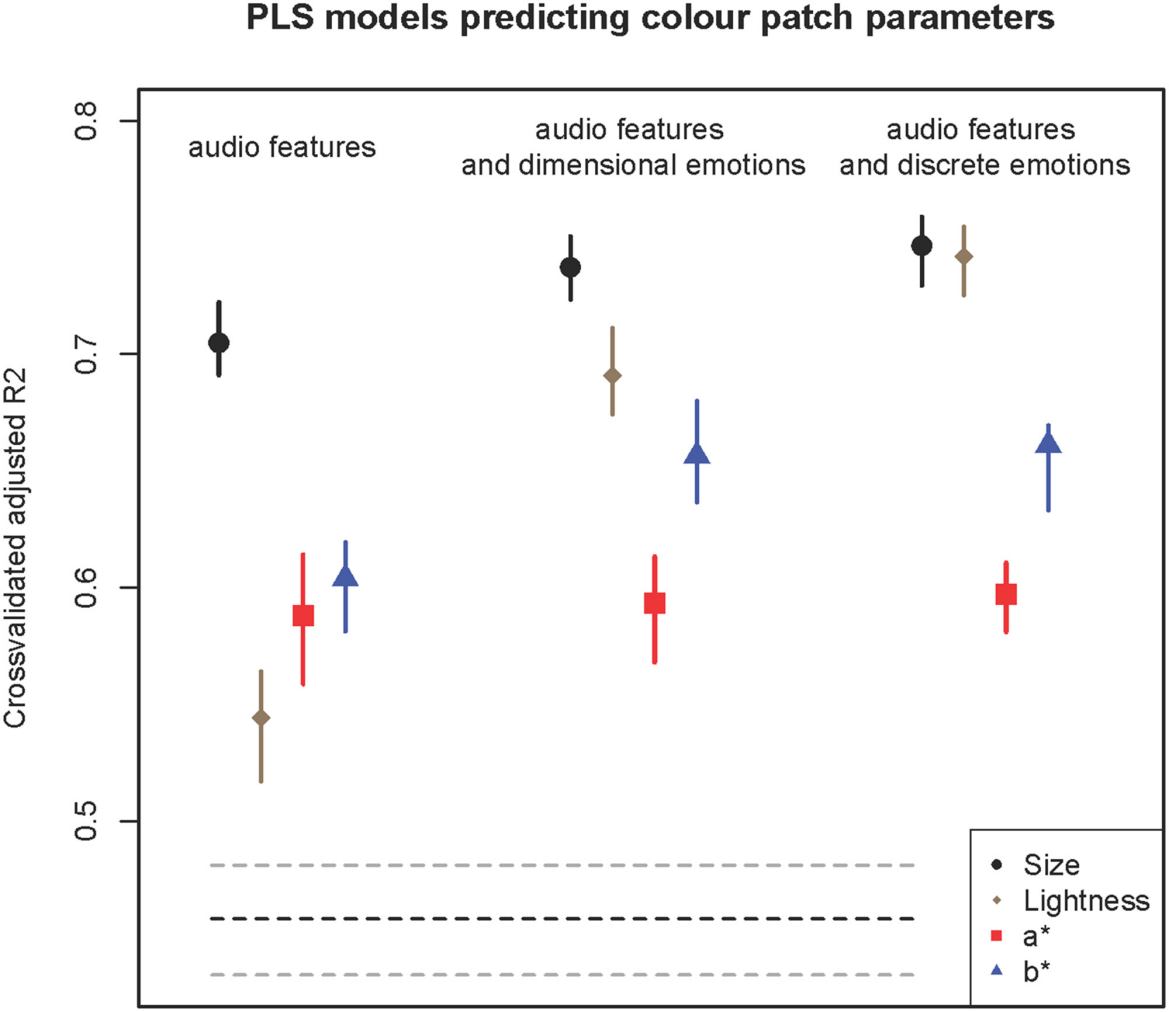
Errorbar plot of variance explained in three models for predicting colour patch parameters. Multivariate predictions of *Size*, *Lightness*, a*, and b* for the three models are given on the x-axis. The level of variance explained, i.e. cross-validated *R* ^*2*^ adjusted for the number of predictors, is given on the y-axis. Symbols indicate the median result in each case with bootstrap 95% confidence interval. The dashed black line at *R* ^*2*^ = 0.458 indicates the noise floor (or chance level), with the two grey dashed lines indicating its 95% confidence interval.

Robust *R* ^*2*^ values (amount of total variance explained), adjusted for the number of predictors, were found by performing cross-validation (in 9 folds, i.e. ‘leave three out’) and taking the median of 5000 repetitions. 95% confidence intervals around the medians were formed using a bootstrap method ([43], following [44], p. 214) with 5000 simulations in each case. We also computed the level of the ‘noise floor’ (i.e., chance level) inherent in the *PLS* method by simulating 5000 randomised inputs to the response interface, calculating the colour patch output, and then producing *R* ^*2*^ with confidence interval on these values in the same way as above. Thus, a model producing *R* ^*2*^ above the noise floor indicates that the corresponding parameters might be successfully predicted. Likewise, the differences in performance between models can be evaluated by comparing medians and confidence intervals. The results for the three models defined above are plotted in Fig 4.

As can be inferred from Table 7 and Fig 4, each of the three models predicted the four colour association parameters well above chance level. In terms of *Size*, the confidence intervals did not overlap. Therefore we can say that the two extended models performed significantly better than the basic Model 1, reaching 75% of variance explained. While the model with audio features was only somewhat successful on its own in predicting *Size* (70%), it was much less successful for the *CIE Lab* parameters, where it explained between 55 and 60% of the total variance. In terms of *Lightness*, the differences between models were striking. The model extended with discrete emotions performed best, predicting 74% of the variance, significantly more than Model 2 (69%) and Model 1 (54%). In terms of b* (blue-to-yellow), the extended models both explained around 66% of the variance, significantly outperforming the basic model. Finally, in terms of a* (green-to-red), all three models explained around 59%. Here, the confidence intervals overlapped, so there was no significant difference between models. Making emotion ratings available to the PLS algorithm did not increase its capacity to explain variation in the a* parameter.

**Table 7:** Three models for multivariate prediction of *Size*, *Lightness*, a*, and b* from audio features with and without emotion ratings.

	*Model 1 (audio)*			*Model 2 (audio & dimensional)*			*Model 3 (audio & discrete)*		
	*R* ^*2*^ cv.	adj. *R* ^*2*^ cv.	conf. int.	*R* ^*2*^ cv.	adj. *R* ^*2*^ cv.	conf. int.	*R* ^*2*^ cv.	adj. *R* ^*2*^ cv.	conf. int.
***Size***	0.728	0.705	0.691…0.722	0.757	0.737	0.723…0.750	0.766	0.746	0.730…0.759
***Lightness***	0.580	0.544	0.517…0.564	0.715	0.691	0.674…0.711	0.762	0.742	0.725…0.754
a*	0.620	0.588	0.559…0.614	0.625	0.593	0.568…0.613	0.629	0.597	0.581…0.611
b*	0.635	0.604	0.581…0.619	0.683	0.657	0.637…0.680	0.688	0.661	0.633…0.669

*R* ^*2*^ cv. = median cross-validated *R* ^*2*^ (amount of total variance explained). adj. *R* ^*2*^ cv. = median cross-validated *R* ^*2*^ adjusted for the number of predictors. conf.int. = 95% confidence interval around median adj. *R* ^*2*^ cv., based on 5000 simulations. For explanation of variable names, see Table 2.

In summary, Models 2 and 3, which were extended to include information on emotion ratings, generally predicted colour parameters better than the basic Model 1, which only had access to audio features. This shows that emotion ratings can function as mediator variables (cf. [38]) when regressing audio features onto colour patch parameters, and lends support to the hypothesis that emotion can be a mediating mechanism for crossmodal correspondences between colour and music.

### Part three: Qualitative analysis of interviews

In order to gain a richer understanding of how people associate colour with sound, the experiment had included a short, structured interview, following methods in [39]. The focus interviews, lasting approximately 10 minutes each (range 6…15 minutes), were organised around four questions: 1) “In your own words, describe your impressions of the experiment”; 2) “How would you describe the way you chose a colour for a sound?”; 3) “What aspects of the colour did you focus on?”; and 4) “What aspects of the sound or music did you focus on?”. The free-form spoken responses were transcribed and carefully analysed. After elimination of filler words, the text corpus consisted of 2585 words, in sentence fragments of between 3 and 18 words in length. The corpus was subjected to classical content analysis (see [40] for a review). A coding frame was set up from the working hypothesis that participants would spontaneously apply informal yet specific listening strategies (cf. [8]) in the crossmodal colour association task. However, such strategies might not be explicit if they were not consciously available to the participants, most of whom were not musically trained. Through a process of bottom-up categorisation of the text corpus, we aimed to detect implicit strategies, and infer their dependence on different crossmodal correspondence mechanisms. Responses where the participant had in some way expressed an association between colour, sound, and emotion, or some other emergent topic, were identified and compared. They were stripped down to fragments highlighting the essentials, and grouped by focal concepts.

#### Associations with emotion

**Fig 5:**
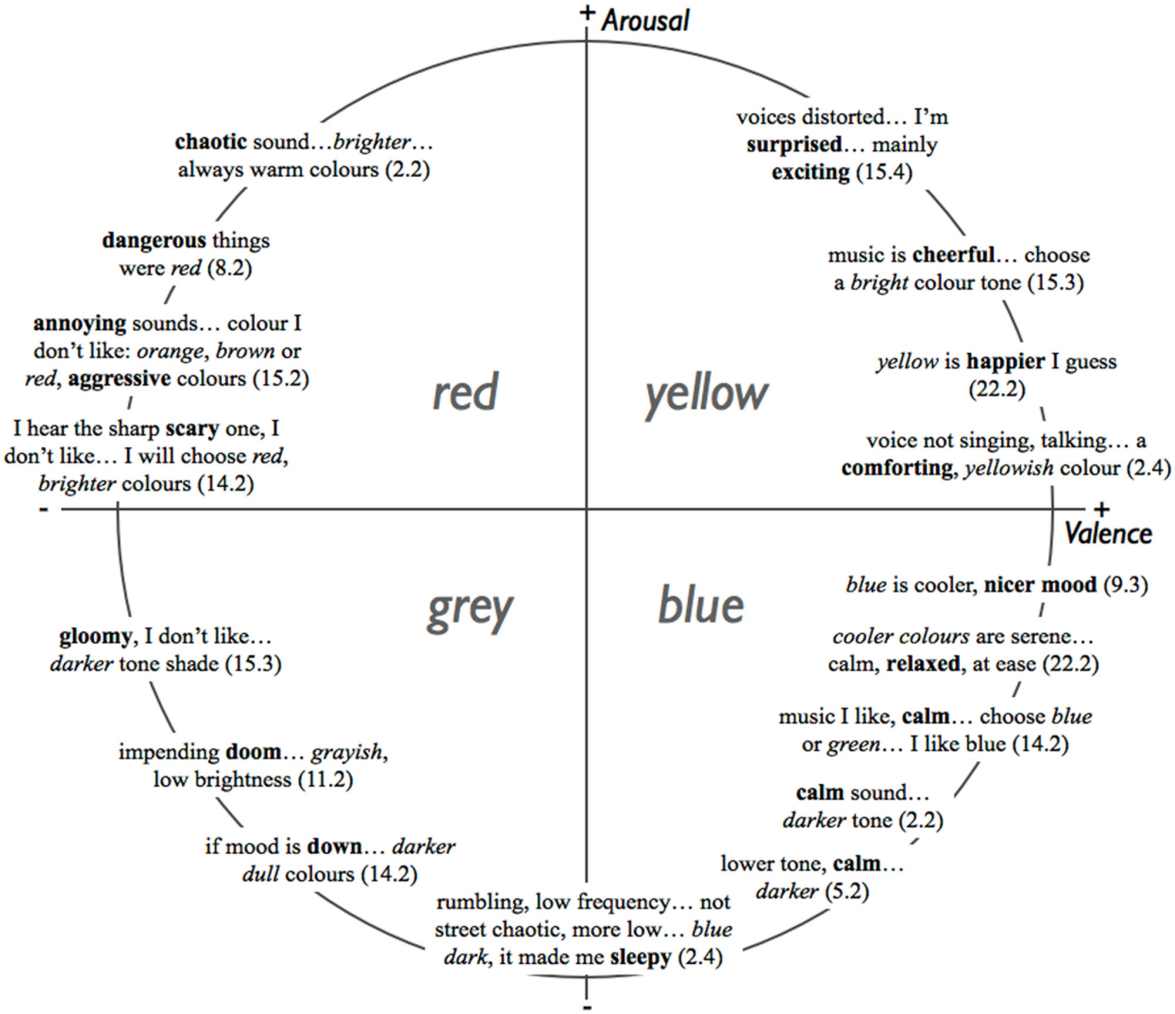
Fragments of spoken interview responses indicating an association between colour and emotion words, distributed in the Valence-Arousal circumplex. Numbers in parentheses refer to *Participant* and *Interview Question*.

We first identified fragments that explicitly related colour or sound with emotion. There were many of these in the corpus, despite the fact that none of the interviewer’s questions directly mentioned emotion (perceived or evoked). After categorisation, selected fragments were laid out in a *Valence (V)—Arousal (A)* circumplex, at the approximate position indicated by a key word indicating affect. A pattern of how colours had been named in association with affect emerged. As Fig 5 shows, fragments mentioning ‘yellow’ appeared in the *V+A+* quadrant (upper right), ‘red’ in the *V-A+* quadrant (upper left), ‘grey’ in the *V-A-* quadrant (lower left), and ‘blue’ or ‘green’ in the *V+A-* quadrant (lower right). Note that the ‘close-to-far’ physical dimension of the interface, i.e. the “y axis” of the Wacom tablet lying flat on the table in front of the participant, was mapped to the b* colour space dimension, i.e. from blue to yellow. The many correspondences between dimensions in colour space (e.g. b*), semantic space (words indicating affect, interpretable in terms of e.g. *Arousal*), the space of dimensional emotions (e.g. perceived *Energy)*, spectral shape (e.g. *Brightness)*, and physical space (i.e. the topology of the response interface) are remarkable. These congruencies, some of which point to SMARC-like effects (cf. [14] and [15]), are likely to depend on more than one crossmodal mechanism, and might be investigated in future work.

#### Associations between colour and sound

**Fig 6:**
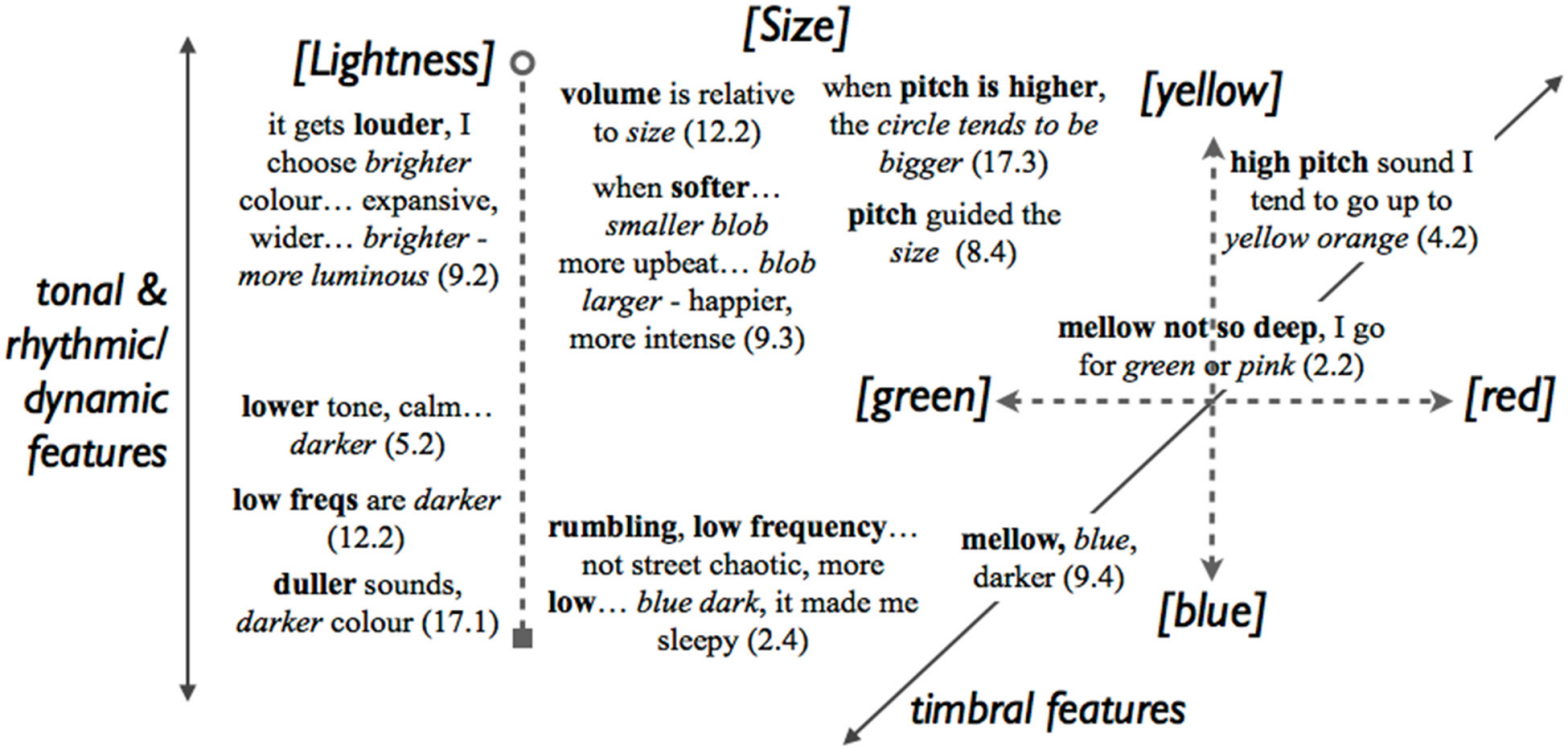
Fragments of spoken interview responses indicating approximate patterns of associations between colour parameters (dotted lines) and audio features (full lines). Numbers in parentheses refer to *Participant* and *Interview Question*.

We then identified the fragments that connected colour and sound. Recall that the unfolding of the emotional mediation mechanism might be described, in Palmer’s words: “as people listen to the music, they have emotional responses… and then pick colors with similar emotional content [as the music]” ([10], p. 3). We reviewed the patterns that had emerged from the correlation analysis (see the previous section and Table 6) in light of the categorised interview responses. In Fig 6, we have attempted to map out the complex relationships between text fragments within a single diagram that is determined by audio features (grouped at the perceptual level) and the colour patch parameters *Size*, *Lightness*, a* (green-to-red), and b* (blue-to-yellow). While this might be a speculative mode of presentation, the layout highlights cases in which the interview response fragments echoed the quantitative results. In particular, the diagram illustrates three patterns that emerged from the correlation analysis in the previous section; first, the correspondence between *Lightness* and tonal features, and inversely, the negative correspondences between *Lightness* and rhythmic/dynamic features; second, the ‘oblique’ (or diagonal) positive correspondence between timbral features and a* and b* dimensions; and third, the correspondences between visual *Size* and sonic “volume” and pitch.

#### Four mechanisms of crossmodal association

Finally, we distilled response fragments that evidenced the different classes of crossmodal association reviewed in the Introduction—structural, ecological, semantic, and emotional—via focal concepts. Through this process, subcategories emerged that were brought together into a tentative framework, shown in Table 8. The leftmost column in the table includes the four hypothesised basic mechanisms, the middle column suggests a ‘link function’ (i.e., the direction of attention) and the rightmost column lists examples of relevant participant response fragments from the present study. Note that several fragments mentioned ‘blue’ in relation to water, and ‘green’ in relation to nature or animals. These are examples, perhaps trivial, of the ecological principle of crossmodal association at work. More subtle is the distinction between, on the one hand, fragments connecting a colour or sound with an actual physical action (e.g. tapping), and, on the other hand, the imagination of being in a physical environment (i.e. imagery or memory). We suggest that the former could include examples of structural crossmodal association, and the latter of emotionally mediated crossmodal association, for the following reason. As it is well known that music can evoke emotions linked with (specific) memories or (prototypical) imagery, it might be that such memory or imagery presents itself to the conscious mind as ‘non-concrete’ entities, or mental representations, alongside the auditory input. The interpretation of such a non-concrete entity, in particular if it contains objects with an ecologically veridical colour, would then strongly influence the way a colour association is made with the music.

**Table 8:** Fragments of spoken interview responses indicating a mechanism of crossmodal association.

*mechanism*	*directed to*	*response fragments indicating crossmodal association*
*structural*	audio spatialisation	*panning demanded zig-zag (6*.*2) left-right… strong panning effect (8*.*2) panning… move left-right (16*.*4)*
*structural*	physical tapping	*rhythm… I tap the pen (16*.*2) giddy-giddy sounds… speccles of colours [makes tapping gesture] (17*.*4)*
*structural*	movement (imagined)	*sound pushing others away (13*.*3) tripping… catch[ing] up (1*.*2) running around (5*.*4)*
*ecological*	water	*blue water (1*.*3*, *21*.*2) water blue (4*.*4*, *5*.*2*, *8*.*2*, *11*.*2*, *16*.*2)*
*ecological*	nature, birds	*nature blueish/greenish (17*.*2) nature green (5*.*2*, *11*.*2) birds green (1*.*3) animals green*, *forest (16*.*2)*
*cognitive / semantic*	tactile feel	*this area [of the colour response interface] warm… muddy… cold (19*.*1)*
*cognitive / semantic*	audiovisual	*noise white (7*.*2) noise vocals machines white (4*.*4) mechanical… gray (21*.*4) singing red (11*.*2)*
*emotional*	memory	*memory childhood (13*.*1)… strong memories (13*.*4) [memories of] walking in Colombia… crowds brownish-red (8*.*3)*
*emotional*	imagery	*I imagine myself on the beach (6*.*2) sound of water…I visualise a beach (21*.*2) film (10*.*4) movie (11*.*2)*

Numbers in parentheses refer to *Participant* and *Interview Question*.

### Summary of results

Summing up and comparing with results from previous studies, we must first caution against broad generalisations given our relatively small sample of participants. At the same time, direct comparisons with previous studies might be unreliable since the reviewed studies used colour schemes in which the parameters are perceptually confounded.

The planned tests revealed that in the present data, *Happy* music was associated with colours of significantly higher *CIE Lightness* than *Anger*, *Fear*, or *Sad* music. This is comparable to previous findings (e.g. [10], p. 2, and [20], p. 3), where music in major keys was rated as more ‘light’ or ‘bright’ than music in minor keys, if we accept the broad assumption that music in the major is generally perceived as more happy. At the same time, *Happy* music was associated with more yellow (rather than blue) colours than *Tender*, *Sad*, or *Fear* music; this replicates and extends findings reported in [10] and [20]. In our study, *Anger* music was associated with more yellow colours than *Sad* or *Tender* music, and, at the same time, with more red (rather than green) colours than *Tender* music. This shows that colour might be used as a response method to identify well-defined discrete emotions. In our study, *Anger* music tended to be associated with large, dark patches towards red and yellow (i.e. ‘terracotta’), replicating findings in [20]. *Tender* music was associated with small, light patches towards red and blue (i.e. ‘violet’). *Happy* music was given large, very light patches towards red and yellow (i.e. ‘orange’). *Fear* and *Sad* music tended to be associated with medium-sized, dark patches towards the middle of the a*-b* plane (i.e. ‘grey’). None of the reviewed previous studies had investigated dimensional emotions. In our study, music of low *Valence* tended to be associated with large, dark colours towards red and yellow (similar to *Anger*), while music of high *Valence* was given small, light patches towards green and blue (similar to *Tender*). Music with low *Energy* was associated with small patches towards green and blue, while high *Energy* music was given large patches towards red and yellow (similar to *Happy*). These findings were consistent with results from the qualitative analysis of focus interviews, which showed that the participants were able to verbalise their colour association strategies, albeit implicitly. This was evidenced, for example, in the connections they spontaneously made between between emotion-words and colour-words, such as ‘danger’ being mentioned together with ‘red’; ‘happy’ with ‘yellow’; ‘gloom’ with ‘grey’; and ‘calm’ with ‘blue’. This compares well with previously reported findings (see [10], Fig 6; and [20]). Finally, the females in this study generally made associations using smaller patches than the males did.

A correlation analysis indicated patterns of relationships between colour patch parameters and audio features. We have interpreted the strong correlations between colour patch *Size* and timbral features of the stimuli as a crossmodal effect at the structural level, whereby music with a brighter and more variable spectrum is perceived as ‘larger’, and thus associated with visual objects of larger size. This might provide an extension of well-known amodal correspondences to physical size, for example with loudness (see [2]), to modal attributes of timbre. The slightly weaker correlations between *Lightness* and tonal or timbral features might be crossmodally mediated by the semantic concept of ‘clarity’. The correspondences between timbral features and both a* and b* were complex and call for further research in order for the patterns that this study has uncovered to be better understood. Comparison with previous work is difficult as none reported computationally extracted audio features. Palmer and collaborators [10] used excerpts of orchestra music with different tempo and reported interesting findings, but in our data, *Tempo* as detected by the *MIR Toolbox* was not significantly associated with any colour patch parameter. Future research might elucidate this matter. Lipscomb and Kim [13] included a factor called “loudness” but it was in fact an amplification percentage on a sampler. Their samples were not normed by a psychoacoustic method, and it cannot be ruled out that their loudness factor was confounded with other audio features such as timbre. In our present work the stimuli were normed, which explains why *Loudness* (N50) did not correlate significantly with any of the colour patch parameters.

We investigated the contribution of emotion to predicting colour patch parameters over and above audio features by performing multivariate linear regressions and comparing three different models. In the basic model, only audio features were available as predictors, while in two extended models, the pool of potential predictors also included discrete and dimensional emotions, respectively. In each model, colour patch parameters were predicted above chance level, and in some cases, up to 75% of the variance was explained. The robustness of the predictive models was evidenced by high cross-validated *R* ^*2*^. Comparison between models revealed that the inclusion of emotion ratings significantly contributed to increased cross-validated *R* ^*2*^ values for three of the colour patch parameters, *Size*, *Lightness*, and, b* (blue-to-yellow), though not for a*. The importance of emotion to the association task was given further support by the qualitative analysis of interviews, which showed that the colour patch decision strategy for several participants was explained or justified by a reference to a visual imagery or episodic memory of a physical situation, which included strong sonic and visual components along with an evoked affect (cf. [7]).

Colour association to music is but one part of the complex research field of crossmodal interactions. As a response modality, colour presents a non-verbal means to understanding how people respond to sound. An important part in the present work was the design of an improved colour response interface. Compared to response methods used in previous published research, ours has certain advantages. Firstly, the interface presents a single colour patch instead of many, thus avoiding visual distraction and confusion. Secondly, the response is fast and continuous, and can be used to track a melodic phrase or dynamic envelope. Thirdly and more generally, using colour as a response modality provides a way to study music perception that does not rely on semantic scales. As Gabrielsson wrote: “Music experience is a complex phenomenon, and is influenced by a variety of interacting factors. Different individuals react differently, and reactions to the same music may vary on different occasions. Many people find it extremely difficult to describe their experience; it seems to elude common vocabulary.” ([45], p. 547). Thus, a ‘free-form’ colour response interface might be useful in music studies with very young children, or with adults of limited semantic capacity such as stroke patients.

## General Discussion

The way people make colour association with music depends on their individual psychological makeup [4], but also, and to a high degree, on the nature of the sound itself; categorical listening strategies might apply. We have seen that some music-colour associations can be explained by neuro-biological mechanisms via structural amodal correspondences [2], by ecological mechanisms via physical source identification [46], and by semantically mediated correspondences via visual imagery [7]. How can emotional mechanisms function in this context? In [46], Gaver proposed that if the perceived input is rich in information, the brain’s task of decoding it is simple. This is the case for source identification within natural environments, where amodal stimulus attributes inferred from different sensory inputs are correlated statistically; this principle might also be fundamental to explaining musical expectancy (cf. [7] and [47]). In a situation of temporary sensory deprivation, however, an organism might still need to correctly infer the physical size of a source to identify a threat. In darkness, the detection of environmental changes depends on accurate sound perception, while in daylight, both visual and sonic inputs are available. The capacity to match input from the two domains—to correctly associate sonic perception with an audiovisual memory of a visually verified physical source—gives a survival advantage. The ecological mechanism is thus produced via statistical correspondences based on natural covariation of sensory attributes. It could then be argued that crossmodal associations based on the principle of ecological perception are independent of emotional processing. Conversely, affectively mediated modal correspondences in humans (and possibly in other higher animals) might arise from the lack of environmental stimulation. In a context of ecological listening, Gaver proposed that in cases where the sensorial input under-specifies a detected event, leaving the source uncertain in the mind of the perceiver, some other mechanism might try to “fill the gap”. He wrote: “If the input for perception is inadequate to specify events, then processing mechanisms must be complex to compensate.” ([46], p. 288). But what happens if higher level processing such as memory recall is still not sufficient? There is experimental evidence that, while sound from an identified source might elicit a predictable affective response, an acoustically equivalent but unidentifiable sound might not do so, or to a lesser extent [48]. Could it be, in such cases, that the ecological crossmodal association mechanism does not kick in? This would suggest that fantasy and imagination could be sparked from incompletely identified events. Interestingly, this mechanism might cause a blurring of the perceived locale of an under-specified event, if ecological and emotional associations contradict each other. We may imagine that, for example, the brain can either determine a ‘source’ of sensory information as residing outside its observing consciousness, as an abstract yet actual entity in the world (e.g., a ghost); or alternatively, it can perceive the ‘source’ as residing inside its mind, as a ‘non-concrete’ entity (e.g., an idea or memory). In either case, it appears the human brain is adept at creating meaningfulness out of thin data, when veridicity escapes it. In this sense, crossmodal associations might emerge from an emotional mechanism that is neither structural nor cognitive, and that works in the opposite way to the ecological principle.

### Limitations and future work

One limitation of the present work lies with the interface design. The motor actions afforded by the physical part of the interface are likely to have limited the responses during the experiment. It is not impossible that the instructions given to the participants before the experiment attenuated individual differences with regard to the range of emotional responses by restricting free visual association and spontaneous imagery that might otherwise have been evoked by the music (cf. [49], p. 9). For example, one person said during the interview: “the sounds were not ‘round’ like the circle, they sound[ed] more dynamic, tense” (participant 2). While this person found that the “interface is intuitive [and] responds well”, others were less comfortable with it: “the pressure determines the shape is another distracting element… single size could be better” (participant 5). It happened on a handful of occasions that a participant would spontaneously move the pen on the Wacom tablet in large sweeping gestures, suggesting that the participant was more preoccupied, at that point, with producing the physical gesture in response to the music, rather than the 'mishmash' of visual colours on the screen. Averaged over time, the response data in such situations would unfortunately simply be more or less ‘grey’, as the interface could not capture the intention behind such gestures. For some participants, the physical gesture of manipulating the interface took on a meaning in itself, beyond that of simply producing a colour patch. For example, one said: “sometimes rhythm beatings, I tap my pen according to the soundfiles” (participant 16), and another highlighted “the rhythms…of scribbling” while making the gesture in the air (participant 9). Could such gestures be coded as categorical responses? Future experimental designs might look into developing the colour response interface further, to account for recognition of gestures. Meanwhile, we are developing a version of the *CIE Lab* response interface for Internet-based experiments. More data will lead to increased analytical power, even if the residual error is expected to be large in view of the reduced control over experimental setting, equipment, and procedure (see [50] for considerations of conducting perceptual research online).

Another limitation of the present work lies in the unresolved conflict or complementarity of the dimensional and discrete emotion models. A possible way forward might be the “hybrid model” of emotion, where “common discrete emotions can be regarded as attractors or hot spots in [a continuous two-dimensional] affect space” ([26], p. 41). This might produce a broad and inclusive model, one that is able to characterise the difference between aesthetic and utilitarian emotions, something which would be suitable for research in multimodal association with the wider world of soundscapes, inclusive of, but vastly larger than, the purely musical.

The present work has shown that when associating colour with music, emotion can function as a mediating mechanism. That is, people associate colour with music in ways that are congruent with the emotions they spontaneously perceive in the music, or with emotions that are connected with memories or imagery within themselves while listening. In future work, we will develop the colour response method further and extend our knowledge of the way crossmodal association works to listening contexts beyond music for film, including, for example, soundscapes, electroacoustic music, and audiovisual performance. We expect all of the structural, ecological, cognitive, and emotional crossmodal association mechanisms to be active in these contexts.
